# Discrimination and Quantification of Glutathione by Cu^+^-Based Nanozymes

**DOI:** 10.3390/bios13080827

**Published:** 2023-08-17

**Authors:** Meixuan Liu, Chen Yan, Qianyun Ye, Xiaohuan Sun, Jie Han

**Affiliations:** School of Chemistry and Chemical Engineering, Yangzhou University, Yangzhou 225002, China; mz120210748@stu.yzu.edu.cn (M.L.); 211004123@stu.yzu.edu.cn (C.Y.); mz120200671@stu.yzu.edu.cn (Q.Y.)

**Keywords:** nanozyme, GSH quantification, GSH discrimination

## Abstract

Glutathione (GSH) is the most abundant low-molecular-weight biological thiol in vivo and has been linked to several diseases. The accurate quantification of GSH is therefore crucial for disease diagnosis and monitoring. In this study, we prepared self-assembled Cu(I)-Cys (cysteine) nanozymes through a two-step procedure. The Cu(I)-Cys nanoparticles exhibited peroxidase-mimicking activity. Upon the addition of H_2_O_2_, they were able to oxidize 3,3,5,5-tetramethylbenzidine (TMB) into oxTMB, resulting in a measurable increase in UV-Vis absorption at 655 nm. However, in the presence of GSH, oxTMB was reduced back to TMB, leading to a decrease in UV-Vis absorption at 655 nm. By utilizing these changes in the absorption intensity, we achieved the sensitive detection of GSH with a detection limit of 2.13 μM. Moreover, taking advantage of the different peroxidase-mimicking activities of Cu(I)-Cys nanoparticles at various pH values, a sensor array with Cu(I)-Cys nanoparticles at pH 4 and pH 5 was constructed. The discrimination of GSH among Cys and ascorbic acid was achieved and the practicability of the sensor array in human serum was validated. This novel approach holds significant promise for the precise discrimination and quantification of GSH and its potential applications in disease diagnosis and therapeutics.

## 1. Introduction

Glutathione (GSH) is the most abundant low-molecular-weight biological thiol in vivo. It is a strong antioxidant and plays significant roles in maintaining the intracellular redox homeostasis [1]. The GSH content has been found to be associated with various diseases, such as Alzheimer’s disease, cancer, and diabetes [2,3,4]. Therefore, precise discrimination and quantification of GSH are crucial. Until now, a series of methods for GSH quantification have been developed, including surface plasmon-coupled emission-related methods [5], electrochemical methods [6], fluorescent methods [7,8,9,10], and colorimetric methods [11,12]. Among these approaches, colorimetric methods offer the advantage of naked-eye detection [13] and hold significant potential for practical GSH-sensing applications.

There are several design principles for colorimetric GSH sensors. Firstly, GSH can react with electrophilic substrates [14]. With an elaborate design, the above process can induce changes in the π-conjugation of the reactant, thereby causing variations in the UV-Vis absorption intensity. Secondly, due to its reducing capability, GSH enables the degradation of specific nanomaterials (e.g., MnO_2_ nanosheets), thereby resulting in a substantial reduction in the UV-Vis absorption [15]. In addition, nanozymes have recently become a hot topic in the field of catalysis [16,17]. Specifically, nanozymes with peroxidase-mimicking activity can produce reactive oxygen species through a reaction with H_2_O_2_ [18,19]. The aforementioned process can be effectively monitored by methylene blue (MB) or 3,3′,5,5′-tetramethylbenzidine (TMB) probes. These probes can be oxidized by reactive oxygen species, resulting in simultaneous changes in both the UV-Vis absorption intensity and solution color [20,21]. However, the presence of GSH can inhibit the above process. Therefore, peroxidase-mimicking nanozymes provide a direct pathway for the construction of colorimetric GSH sensors. Although GSH quantification has been achieved with high sensitivity using the above methodology, the precise discrimination of GSH has not been paid enough attention.

Herein, a two-step procedure was employed to construct self-assembled Cu(I)-Cys (cysteine) nanozymes. The prepared Cu(I)-Cys nanozymes demonstrated peroxidase-mimicking activity. In the presence of H_2_O_2_, Cu(I)-Cys nanoparticles oxidized TMB, resulting in the formation of blue-colored oxTMB and an increase in the UV-Vis absorption at 655 nm. However, in the presence of an antioxidant, oxTMB was reduced to TMB, causing a decrease in the UV-Vis absorption at 655 nm. By leveraging the changes in the UV-Vis absorption intensity, sensitive detection of GSH with a detection limit of 2.13 μM was achieved. Furthermore, taking advantage of the various peroxidase-mimicking activities of the Cu(I)-Cys nanozymes at different pH values, a sensor array with a precise GSH discrimination capability was constructed and its potential application in human serum was demonstrated.

## 2. Materials and Methods

Materials: CuCl_2_ and Cys were provided by Macklin Biochemical Technology Co., Ltd. (Shanghai, China). GSH was purchased from Aladdin Biochemical Technology Co., Ltd. (Shanghai, China). Human serum was purchased from Solarbio science & technology Co., Ltd. (Beijing, China). The other reagents were provided by Sinopharm Chemical Reagent Co., Ltd. (Shanghai, China). The deionized water was filtered through a Milli-Q Plus system (Millipore, Molsheim, France).

Instruments: The morphology of the as-prepared samples was investigated by an SEM (Zeiss Supra55, Zeiss, Oberkochen, German) and HRTEM (Tecnai G2 F30 S-Twin TEM, FEI, Hillsboro, OR, USA). The ultraviolet-visible (UV-Vis) spectra were obtained using a Cary 5000 spectrometer (Varian, Palo Alto, CA, USA). Equipped with standard monochrome Al Kα, the phase composition was measured using an Axis Ultra X-ray photoelectron spectrometer (XPS, Kratos Analytical, Manchester, UK) with a source (hv = 1486.6 eV). The fluorescent study was carried out using a Fluorescence Spectrometer F-7000 (Hitachi, Tokyo, Japan). The zeta-potential was measured with a ZEN3690 zetasizer (Malvern, Malvern, UK).

Preparation of Cu(II)-Cys nanoparticles: The Cu(II)-Cys nanoparticles were prepared according to a previous publication [22]. Specifically, Cys (2 mmol) and NaOH (2 mmol) were dissolved in water (10 mL). Then, the above mixture was added to an aqueous solution of CuCl_2_ (10 mL). After 5 min, the Cu(II)-Cys nanoparticles were collected by centrifugation and washed with water and ethanol.

Preparation of Cu(I)-Cys nanoparticles: The obtained Cu(II)-Cys nanoparticles were mixed with GSH (50 mM) under N_2_ atmosphere. After stirring for 2 h, the Cu(I)-Cys nanoparticles were collected by centrifugation and washed with water. 

Quantification of GSH with Cu(I)-Cys nanoparticles: The GSH concentration was quantified by taking advantage of the change in the UV-Vis absorption intensity at 655 nm. Specifically, TMB (0.5 mM), H_2_O_2_ (5 mM), and Cu(I)-Cys nanoparticles (100 μg/mL) were mixed in buffer solution (pH = 4). The above mixture was left to react for 80 min at 37 °C. Subsequently, various concentrations of GSH were added and the corresponding UV-Vis absorption spectra were recorded. Using the above method, the detection limit of GSH was calculated based on the equation of LOD = 3 σ/k, where σ represents the standard deviation of the UV-Vis absorption response, which was calculated as 0.00744, and k represents the slope of the calibration curve.

Construction of the sensor array for the determination of GSH, Cys, and AA: TMB (0.5 mM), H_2_O_2_ (5 mM), and Cu(I)-Cys nanoparticles (100 μg/mL) were mixed at pH = 4 and pH = 5. After 80 min of reaction, GSH, Cys, or AA at specific concentrations were added into the above mixture and the UV-Vis intensity at 655 nm was recorded. Five repetitions were conducted for each experiment to obtain the 2 sensors × 3 analytes × 5 replicates data matrix. The data matrix was further analyzed using the linear discriminant analysis (LDA) methodology. 

## 3. Results and Discussion

**Figure 1:**
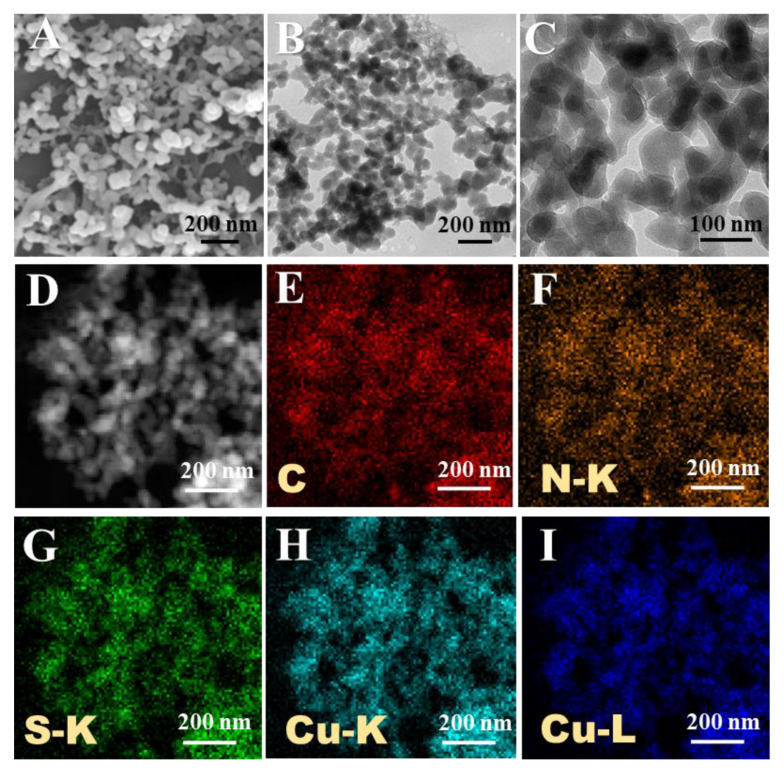
(**A**) SEM image, (**B**,**C**) TEM image, and (**D**) HAADF-STEM image of Cu(I)-Cys nanoparticles. EDX mapping of the (**E**) C, (**F**) N, (**G**) S, and (**H**,**I**) Cu elements of Cu(I)-Cys nanoparticles.

**Figure 2:**
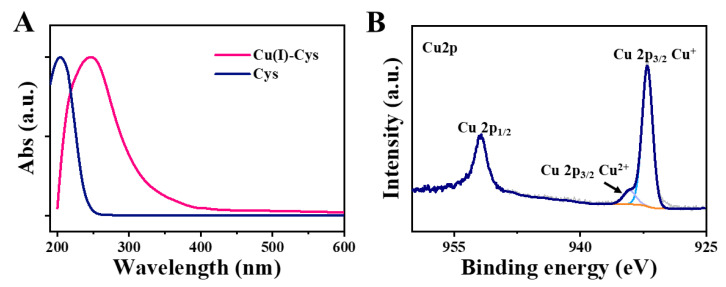
(**A**) UV-Vis spectra of Cys and Cu(I)-Cys nanoparticles. (**B**) XPS Cu 2p spectrum of Cu(I)-Cys nanoparticles.

The Cu(II)-Cys nanoparticles were prepared through the coordination interaction between Cu^2+^ and Cys [22]. Subsequently, the Cu(II)-Cys nanoparticles were mixed with GSH for 2 h. After thorough purification, Cu(I)-Cys nanoparticles were obtained. The morphology of the Cu(I)-Cys nanoparticles was then investigated. As shown in Figure 1, from both the SEM and TEM images (Figure 1A–C), a homogeneous distribution of spherical nanoparticles with an average diameter of ~70 nm was observed. After further inspection of the HAADF-STEM (high-angle annular dark-field scanning TEM) image and EDX (energy-dispersive X-ray spectroscopy) mapping results (Figure 1D–I), an even distribution of the C, N, S, and Cu elements was observed, indicating that the Cu(I)-Cys nanoparticles were composed of both copper ions and Cys. In addition, the UV-Vis spectra of the Cys and Cu(I)-Cys nanoparticles were recorded. From Figure 2A, it is obvious that the maximum absorption wavelength of Cys shifted from 204 nm to 247 nm, suggesting an interaction between the copper ion and Cys. Moreover, from the XPS spectra (Figure S1 in Supplementary Materials), peaks corresponding to S 2p, C 1s, N 1s, O 1s, and Cu 2p were found. This result further confirmed the composition of the Cu(I)-Cys nanoparticles. After further analysis of the Cu 2p spectrum (Figure 2B), the peak with a binding energy of 952.03 eV was ascribed to Cu 2p1/2 and the peaks with a binding energy of ~932 eV were ascribed to Cu 2p3/2. In particular, the peak with a strong intensity at 932.13 eV was assigned to Cu(I) and the broad peak with a low intensity at 932.61 eV was assigned to Cu(II). The ratio of Cu(I)/Cu(II) was determined to be 6.38, suggesting that the copper ions in the Cu(I)-Cys nanoparticles mainly existed in the form of Cu(I). Given that the Cu(II)-Cys nanoparticles were treated with GSH, which is a known reducing agent, it is reasonable that the original Cu(II) was mostly reduced to Cu(I) by GSH. In addition, the zeta-potential of the Cu(I)-Cys nanoparticles was demonstrated to be −14 mV (Figure S2), suggesting good colloidal stability.

According to a previous publication, Cu(I) can react with H_2_O_2_ and generate ^.^OH through the Fenton reaction [23]. Therefore, it was assumed that the Cu(I)-Cys nanoparticles exhibited peroxidase-mimicking activity. To confirm the above assumption, a DCFH (2,7-dichlorodihydrofluorescein) probe was employed. It is well known that DCFH is a non-fluorescent compound. However, upon oxidation by reactive oxygen species, it can be converted to DCF (2,7-dichlorofluorescein), resulting in the emission of fluorescence at ~520 nm [24]. As shown in Figure S3A, the individual DCFH solution and the mixture of DCFH and H_2_O_2_ showed rather weak fluorescence. However, when mixed with both H_2_O_2_ and Cu(I)-Cys nanoparticles, a highly emissive fluorescence was observed in the DCFH aqueous solution. The above result suggests that the Cu(I)-Cys nanoparticles exhibited peroxidase-mimicking activity. To confirm the above conclusion, MB was used as an additional probe to assess the enzymatic catalytic activity of the Cu(I)-Cys nanoparticles. As shown in Figure S3B, MB demonstrated maximum UV-Vis absorption at 652 nm. In the presence of H_2_O_2_, the UV-Vis absorption of MB barely changed. However, with the co-existence of H_2_O_2_ and Cu(I)-Cys nanoparticles, the UV-Vis absorption of MB at 652 nm gradually decreased along with time (Figure S4) and almost completely disappeared within 60 min. Taken together, it is safe to say that the Cu(I)-Cys nanoparticles prepared in this study demonstrated peroxidase-mimicking activity and can be regarded as nanozymes. 

**Figure 3:**
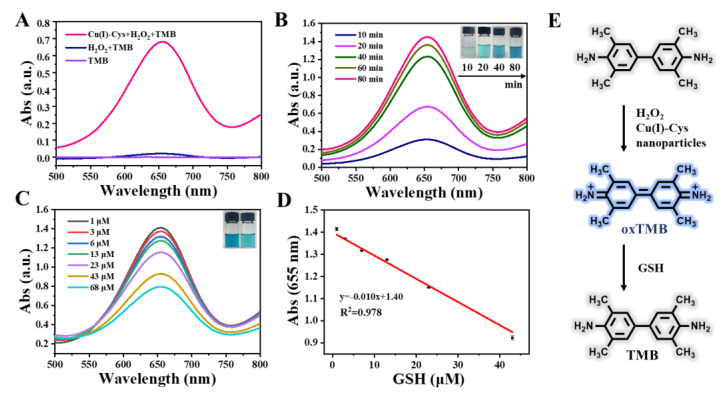
(**A**) UV-Vis spectra of TMB, TMB + H_2_O_2_, and TMB + H_2_O_2_ + Cu(I)-Cys nanoparticles. The concentrations of TMB, H_2_O_2_, and Cu(I)-Cys nanoparticles were kept at 0.5 mM, 5 mM, and 100 μg/mL, respectively. Reaction time: 15 min. Temperature: 37 °C. pH: 4. (**B**) UV-Vis spectra of TMB + H_2_O_2_ + Cu(I)-Cys nanoparticles as a function of reaction time. Inset: Photos of the TMB + H_2_O_2_ + Cu(I)-Cys nanoparticle mixture at various reaction times. (**C**) UV-Vis spectra of TMB + H_2_O_2_ + Cu(I)-Cys nanoparticles in the presence of various concentrations of GSH. Inset: Photos of the TMB + H_2_O_2_ + Cu(I)-Cys nanoparticle mixture before (left) and after the addition of GSH (right). (**D**) The calibration curve of GSH obtained from C. (**E**) Schematic representation of the sensing principle of GSH using the TMB + H_2_O_2_ + Cu(I)-Cys nanoparticle-related sensing platform.

TMB is another commonly used substrate for studying nanozymes with peroxidase activity [25]. It is known that the aqueous solution of TMB is colorless and shows negligible UV-Vis absorption at around 650 nm. When oxidized to oxTMB by reactive oxygen species, its color turns blue and a significant increase in the UV-Vis absorption at ~650 nm is observed. The results shown in Figure 3A,B provide clear evidence that the Cu(I)-Cys nanoparticles catalyzed the oxidization of TMB in the presence of H_2_O_2_. At pH 4, the UV-Vis absorption significantly increased within 20 min and the color of the above mixture turned evidently blue. Given that the above UV-Vis absorption and color change were induced by the Cu(I)-Cys nanoparticles + H_2_O_2_-triggered oxidation, the presence of an antioxidant may inhibit the above process, resulting in a decrease in the UV-Vis intensity and fading of the blue color. With the above idea in mind, GSH, one of the abundant reducing agents in vivo, was employed to confirm the above assumption. To investigate the effect of GSH, after 80 min of reaction of the Cu(I)-Cys nanoparticles, H_2_O_2_, and TMB, the UV-Vis absorption spectra of the above mixture in the presence of various concentrations of GSH were recorded. From Figure 3C, it is evident that with the increase in the GSH concentration, the UV-Vis absorption at 655 nm gradually decreased. As depicted in the inset of Figure 3C, the blue color of oxTMB obviously faded. The above results demonstrate that the presence of GSH can reduce oxTMB to TMB. Interestingly, after plotting the UV-Vis absorption intensity at 655 nm against the GSH concentration, a linear calibration curve for GSH was obtained (Figure 3D). Taken together, by manipulating the Cu(I)-Cys nanoparticles-initiated enzymatic process, GSH can be quantified through simple measurement of the UV-Vis absorption intensity (Figure 3E). Therefore, the mixing of Cu(I)-Cys nanoparticles with H_2_O_2_ and TMB can be considered as a sensing platform for GSH. Furthermore, taking advantage of the commonly used tripling signal-to-noise ratio principle, using the above method, the detection limit of GSH was calculated to be 2.13 μM. Although the detection limit is a bit higher than the reported surface-enhanced Raman spectroscopy [26] and mass spectroscopy [27] methodologies, it is quite low for colorimetric methods according to the previous literature [28]. 

**Figure 4:**
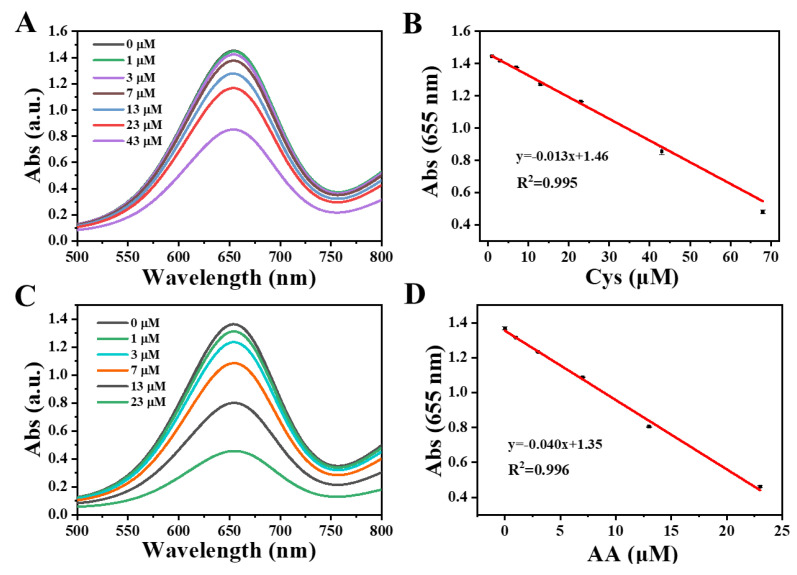
(**A**) UV-Vis spectra of TMB + H_2_O_2_ + Cu(I)-Cys nanoparticles in the presence of various concentrations of Cys at pH 4. (**B**) The calibration curve of Cys obtained from A. (**C**) UV-Vis spectra of TMB + H_2_O_2_ + Cu(I)-Cys nanoparticles in the presence of various concentrations of AA at pH 4. (**D**) The calibration curve of AA obtained from C.

Besides sensitivity, selectivity is also an important factor in the evaluation of sensors. To investigate the selectivity of the Cu(I)-Cys nanoparticles-based GSH sensor, glutamate (Glu), glycine (Gly), alanine (Ala), valine (Val), threonine (Thr), serine (Ser), phenylalanine (Phe), histidine (His), lysine (Lys), Cys, maltose, galactose, glucose, Na^+^, K^+^, Ca^2+^, urea, thiourea, bovine serum albumin (BSA), an oxidized form of GSH (GSSG), and common antioxidant (ascorbic acid, AA) were used as interferents. Similarly, after 80 min of reaction of the Cu(I)-Cys nanoparticles, H_2_O_2_, and TMB, the UV-Vis absorption intensity at 655 nm of the above mixture in the presence of various interferents was recorded. As shown in Figure S5, no obvious decrease in the UV-Vis absorption was observed when Glu, Gly, Ala, Val, Thr, Ser, Phe, His, Lys, maltose, galactose, glucose, Na^+^, K^+^, Ca^2+^, urea, thiourea, BSA, and GSSG were employed, indicating their negligible interference in the detection of GSH. However, in the case of Cys and AA, an evident decrease in the UV-Vis absorption was obtained. Since both Cys and AA are known as reducing agents, the results acquired are reasonable. After plotting the UV-Vis absorption at 655 nm against the concentration of Cys and AA (Figure 4A,C), the calibration curves for Cys and AA (Figure 4B,D) were obtained as well. From the calibration curves of GSH, Cys, and AA, we can see that one of the AA curves is significantly different from those of GSH and Cys, which allows for the easy discrimination of AA from GSH and Cys. After careful inspection of the calibration curves of GSH and Cys, one can find that the calibration curves of GSH and Cys are quite similar, although the slope of GSH is slightly larger than that of Cys.

**Figure 5:**
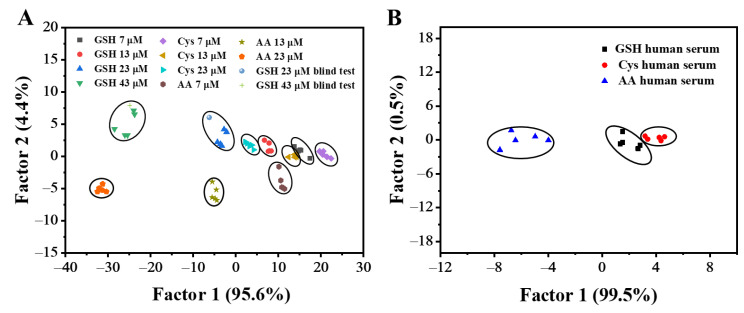
(**A**) LDA plot for the discrimination of GSH, Cys, and AA at different concentrations using a Cu(I)-Cys nanoparticles-based sensor array. (**B**) LDA plot for the discrimination of GSH, Cys, and AA at 10 μM in 10% human serum using a Cu(I)-Cys nanoparticles-based sensor array.

According to the above results, the discrimination of GSH among Cys and AA may be difficult when using the Cu(I)-Cys nanoparticles + H_2_O_2_ + TMB sensing platform at pH 4. To improve the discriminant capability, the construction of a sensor array was assumed to be an effective methodology due to its ability to collect multivariate data. Considering that pH is a crucial factor that influences the activity of nanozymes, the peroxidase-mimicking activity of the Cu(I)-Cys nanoparticles was assumed to differ at various pH values, which may induce different patterns of UV-Vis absorption for GSH, Cys, and AA. To confirm this assumption, the UV-Vis absorption spectra of the Cu(I)-Cys nanoparticles + H_2_O_2_ + TMB sensing platform in the presence of various concentrations of GSH, Cys, and AA at pH 5 (Figure S6A–C) were recorded. By plotting the UV-Vis absorption intensity at 655 nm against the concentration, the calibration curves of GSH, Cys, and AA at pH 5 (Figure S6D–F) were obtained. In this case, we found that the slope of the calibration curve of GSH was slightly smaller than that of Cys. Although the difference in the UV-Vis absorption may not be sufficient for the discrimination of GSH and Cys at a specific pH, by integrating the results obtained at pH 4 and 5, the difference between them is enlarged. Therefore, the Cu(I)-Cys nanoparticles + H_2_O_2_ + TMB sensing platform at pH 4 and pH 5 was employed for the construction of a two-sensor-composed sensor array. For each sensor, after 80 min of reaction of the Cu(I)-Cys nanoparticles, H_2_O_2_, and TMB, the UV-Vis absorption at 655 nm was monitored in the presence of 23 μM GSH, Cys, or AA. Five repetitions were conducted to obtain the 2 sensors × 3 analytes × 5 replicates data matrix. The data matrix was then analyzed using the LDA methodology. LDA is a supervised dimensionality reduction statistical methodology. In an LDA plot, tight clustering of each component separated by a large intercluster distance reveals an excellent discrimination capability. As shown in Figure S7, well-separated clusters were observed for GSH, Cys, and AA. The clear boundaries observed between different clusters indicated the excellent discrimination capability of the as-designed sensor array. To allow for further discrimination of GSH, Cys, and AA at different concentrations, the data matrixes of GSH (7 μM, 13 μM, 23 μM, and 43 μM), Cys (7 μM, 13 μM, and 23 μM), and AA (7 μM, 13 μM, and 23 μM) were collected by taking advantage of the Cu(I)-Cys nanoparticles-based sensor array and subsequently analyzed using the LDA method. As shown in Figure 5A, different concentrations of GSH, Cys, and AA showed well-separated tight clusters, confirming that the Cu(I)-Cys nanoparticles-based sensor array enables the differentiation of antioxidants of different types and concentrations. 

To further prove the accuracy of the quantitative discrimination of the antioxidants, blind tests with 23 μM and 43 μM of GSH were performed. For each blind test, the UV-Vis absorption intensity of the TMB + H_2_O_2_ + Cu(I)-Cys nanoparticles + GSH at pH 4 and 5 was recorded and subsequently used for the LDA analysis. As shown in Figure 5A, the samples with a GSH concentration of 23 μM and 43 μM were correctly classified to their corresponding clusters, indicating the high discrimination accuracy of the sensor array. 

To prove the potential of the practical application of the as-designed sensor array, we conducted antioxidant sensing experiments in 10% human serum. By analyzing the data matrixes (3 analytes × 5 replicates) collected at both pH 4 and 5 using the LDA methodology, the separated clusters representing GSH, Cys, and AA were observed in the LDA plot (Figure 5B). The clear boundaries between the different clusters reveal the discriminant capability of the Cu(I)-Cys nanoparticles-based sensor array. Taken together, the above results indicate the practicability of the discrimination of GSH, Cys, and AA using the Cu(I)-Cys nanoparticles-based sensor array. 

## 4. Conclusions

In conclusion, we report a novel approach for the sensitive quantification and precise discrimination of GSH using Cu(I)-Cys nanoparticles. The Cu(I)-Cys nanoparticles were demonstrated to have peroxidase-mimicking activity. In the presence of H_2_O_2_, the Cu(I)-Cys nanoparticles were imparted with the capability to oxidize TMB to oxTMB. By inhibiting the above process, the antioxidant GSH can be quantified by the UV-Vis absorption variation. The detection limit of GSH was calculated to be as low as 2.13 μM. Moreover, taking advantage of the different peroxidase-mimicking activities of the Cu(I)-Cys nanoparticles at various pH values, a sensor array was constructed and the precise discrimination of GSH among Cys and AA was achieved. Moreover, the practicability of the Cu(I)-Cys nanoparticles-based sensor array in human serum was validated. It is believed that the current work provides direct guidance for the practical application of GSH sensors. 

## Data Availability

Data will be made available on request.

## Supplementary Materials

The following supporting information can be downloaded at: https://www.mdpi.com/article/10.3390/bios13080827/s1. Figure S1: XPS spectrum of Cu(I)-Cys nanoparticles; Figure S2: Zeta-potential of Cu(I)-Cys nanoparticles; Figure S3: Fluorescence spectra of DCFH, DCFH + H_2_O_2_, and DCFH + H_2_O_2_ + Cu(I)-Cys nanoparticles and the UV-Vis spectra of MB, MB + H_2_O_2_, and Cu(I)-Cys nanoparticles; Figure S4: UV-Vis spectra of MB + H_2_O_2_ +Cu(I)-Cys nanoparticles as a function of reaction time; Figure S5: UV-Vis absorption intensity (655 nm) of TMB + H_2_O_2_ + Cu(I)-Cys nanoparticles in the presence of Glu, Gly, Ala, Val, Thr, Ser, Phe, His, Lys, Cys, maltose, galactose, glucose, Na^+^, K^+^, Ca^2+^, urea, thiourea, BSA, GSSG, GSH, and AA; Figure S6: UV-Vis absorption spectra of TMB + H_2_O_2_ + Cu(I)-Cys nanoparticles in the presence of various concentrations of GSH, Cys, and AA and their corresponding calibration curves; Figure S7: LDA plot for the discrimination of GSH, Cys, and AA at 23 μM using a Cu(I)-Cys nanoparticles-based sensor array.
