# Supplementary material for: Discrimination and Quantification of Glutathione by Cu+-Based Nanozymes
Source: Biosensors (Basel). 2023 Aug 17;13(8):827. doi: 10.3390/bios13080827 (PMC10452140; doi:10.3390/bios13080827)
Supplement: Supplementary file 1 [file biosensors-13-00827-s001.zip › biosensors-2528749-supplementary.pdf]

Discrimination and Quantification of Glutathione by Cu^+-Based Nanozymes

Meixuan Liu †, Chen Yan †, Qianyun Ye, Xiaohuan Sun * and Jie Han *

School of Chemistry and Chemical Engineering, Yangzhou University, Yangzhou 225002, China; mz120210748@stu.yzu.edu.cn (M.L.); 211004123@stu.yzu.edu.cn (C.Y.); mz120200671@stu.yzu.edu.cn (Q.Y.)

* Correspondence: xhuansun@yzu.edu.cn (X.S.); hanjie@yzu.edu.cn (J.H.).

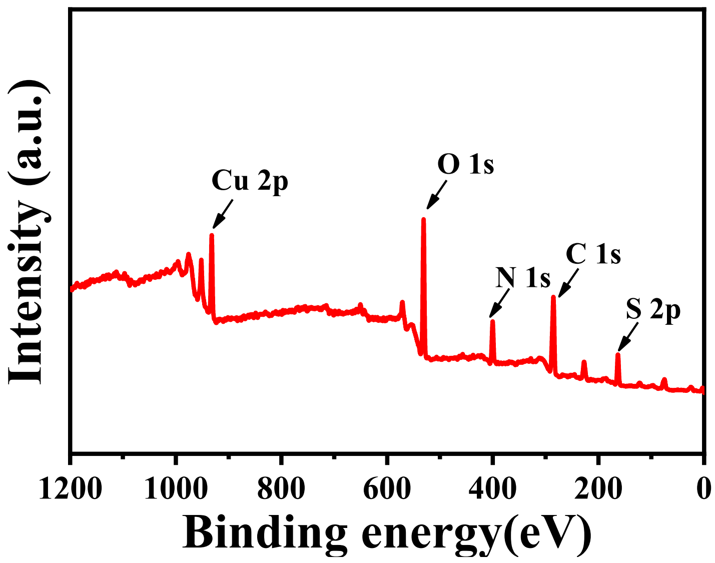

Figure S1. The XPS spectrum of Cu(I)-Cys nanoparticles.

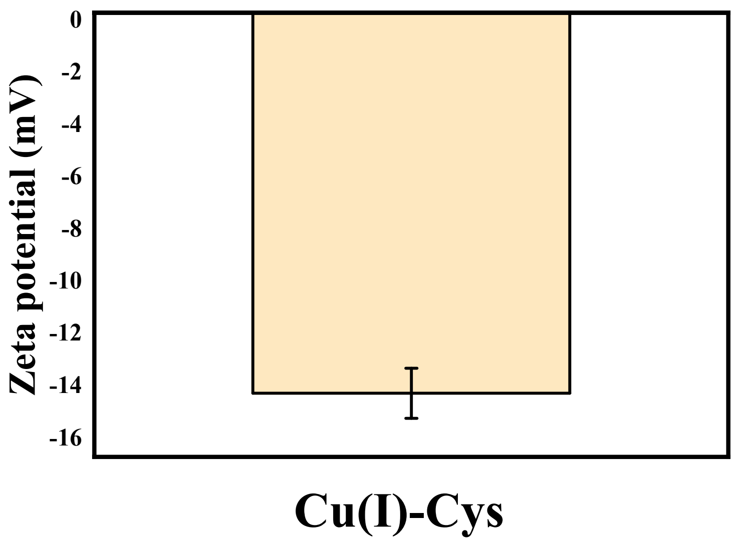

Figure S2. Zeta-potential of Cu(I)-Cys nanoparticles.

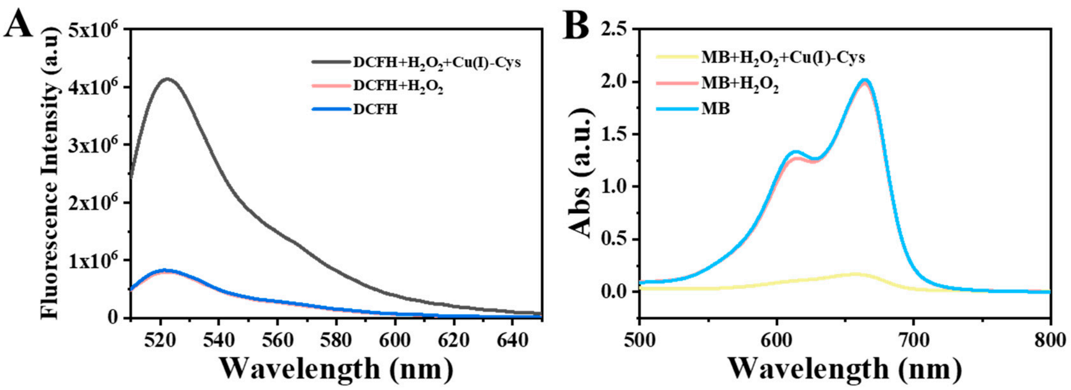

Figure S3. (A) The fluorescence spectra of DCFH, DCFH+H_2O_2 and DCFH+H_2O_2+Cu(I)-Cys nanoparticles. The concentration of DCFH, H_2O_2 and Cu(I)-Cys nanoparticles was kept at 50 μg/mL, 50 mM and 100 μg/mL, respectively. Reaction time: 5 min. (B) The UV-Vis spectra of MB, MB+H_2O_2 and Cu(I)-Cys nanoparticles. The concentration of MB, H_2O_2 and Cu(I)-Cys nanoparticles was kept at 10 μg/mL, 50 mM and 100 μg/mL, respectively. Reaction time: 90 min.

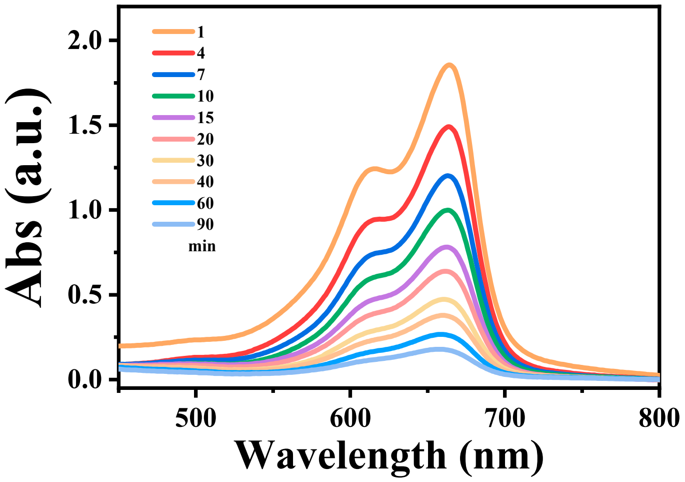

Figure S4. The UV-Vis spectra of MB+H_2O_2 +Cu(I)-Cys nanoparticles as a function of reaction time. The concentration of MB, H_2O_2 and Cu(I)-Cys nanoparticles was kept at 10 μg/mL, 50 mM and 100 μg/mL.

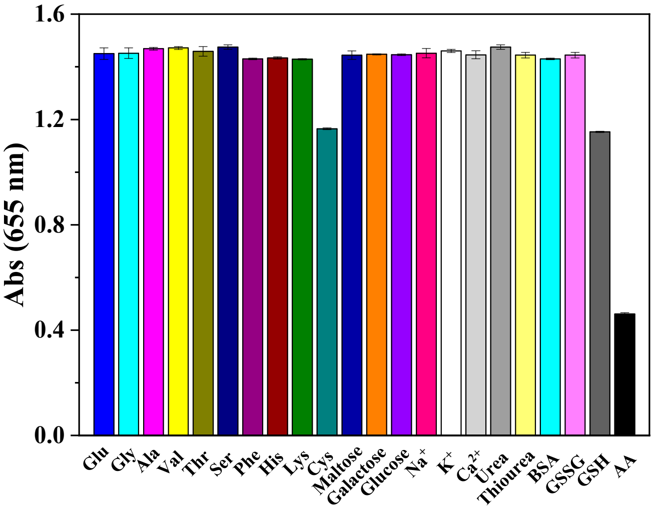

Figure S5. The UV-Vis absorption intensity (655 nm) of TMB+H_2O_2+Cu(I)-Cys nanoparticles in the presence of Glu, Gly, Ala, Val, Thr, Ser, Phe, His, Lys, Cys, maltose, galactose, glucose, Na^+, K^+, Ca^{2+}, Urea, thiourea, BSA, GSSG, GSH and AA at pH 4. The concentration of BSA was kept at 25 µg/mL and the concentration of all the other analytes was kept at 25 µM.

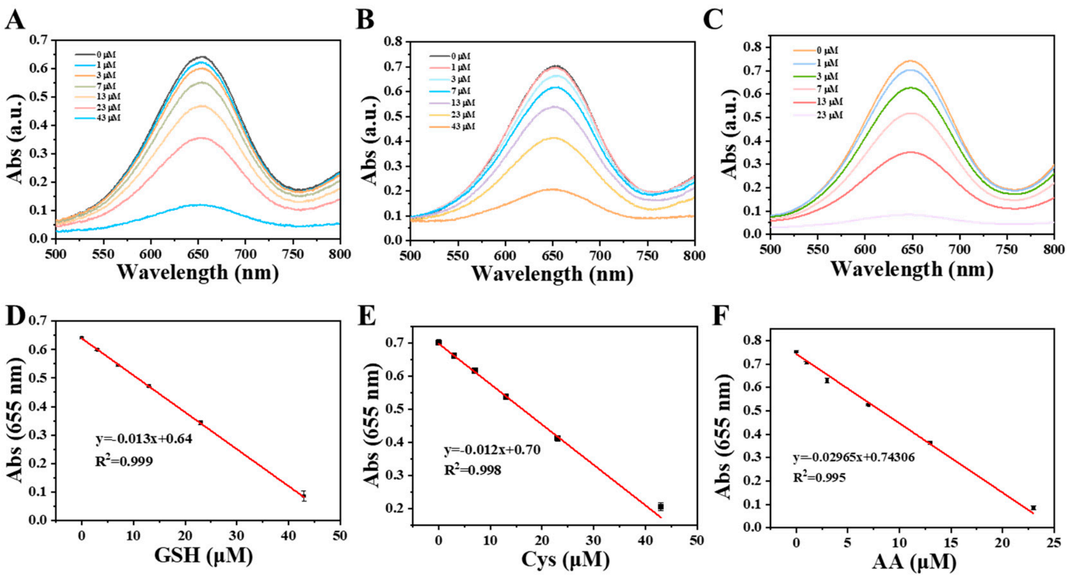

Figure S6. The UV-Vis spectra of TMB+H_2O_2+Cu(I)-Cys nanoparticles in the presence of various concentrations of (A) GSH, (B) Cys and (C) AA at pH 5. The calibration curve of (D) GSH, (E) Cys and (F) AA at pH 5.

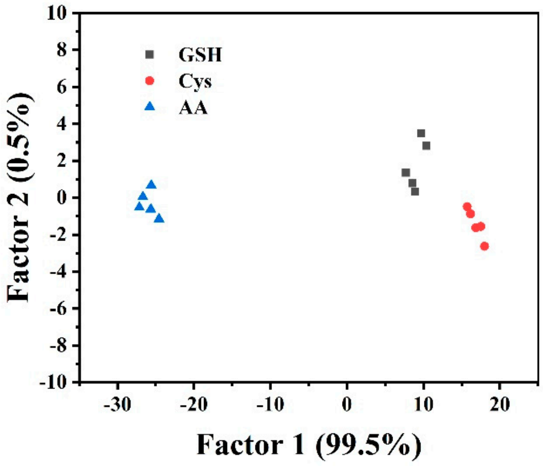

Figure S7. The LDA plot for the discrimination of GSH, Cys and AA at 23 μM using a Cu(I)-Cys nanoparticles-based sensor array.
